# Measuring progress in availability and readiness of Basic emergency obstetric and newborn care (BEmONC) services in Bangladesh, 2014–2017

**DOI:** 10.1371/journal.pone.0314116

**Published:** 2025-02-14

**Authors:** Abu Sayeed, Nondo Saha, Ema Akter, Md. Mehedi Hasan, Anita Pickard, Anindita Saha, Abu Bakkar Siddique, Aniqa Tasnim Hossain, Shafiqul Ameen, Sabrina Jabeen, Tanjeena Tahrin Islam, Lubna Hossain, Sahar Raza, K. M. Tanvir, Fariya Rahman, Farhana Jahan, Mohammed Ahsanul Alam, Shams El Arifeen, Ahmed Ehsanur Rahman, Anisuddin Ahmed

**Affiliations:** 1 Maternal and Child Health Division (MCHD), International Centre for Diarrhoeal Disease Research, Bangladesh (icddr,b), Dhaka, Bangladesh; 2 Poche Centre for Indigenous Health, The University of Queensland, Toowong, Queensland, Australia; 3 School of Public Health, University of Sydney, Sydney, NSW, Australia; 4 Institute of Statistical Research and Training, University of Dhaka, Dhaka, Bangladesh; 5 Department of Public Health, North South University, Dhaka, Bangladesh; 6 National Institute of Population Research and Training (NIPORT), Dhaka, Bangladesh; 7 Global Health and Migration Unit, Department of Women’s and Children’s Health, Uppsala University, Uppsala, Sweden; African Population and Health Research Center, KENYA

## Abstract

Increasing the availability and readiness of basic emergency obstetric and newborn care (BEmONC) services is essential for improving maternal and neonatal health. However, little is known about any progress made in the availability and readiness of BEmONC services in Bangladesh. Using nationally representative data from the Bangladesh Health Facility Survey conducted between 2014 and 2017, we measured changes in the availability and readiness of BEmONC services in health facilities in Bangladesh, calculating the BEmONC service availability and readiness scores according to the World Health Organization Service Availability and Readiness Assessment guideline. The percentage of health facilities performing all seven basic signal functions declined slightly from 13% in 2014 to 11% in 2017. The decline was largely noticed in Maternal and Child Welfare Centers, Upazila Health Complexes, and Union Subcenter/Rural Dispensaries, as well as in all divisions except Rangpur. No remarkable changes in overall readiness of health facilities across location, division and facility type were observed between 2014 and 2017. However, significant reductions in availability and readiness were noticed when item-specific assessment was made. Type of health facility was significantly associated with both availability and readiness scores in adjusted regression models. Appropriate strategies and efforts could improve the availability and readiness of BEmONC services in health facilities in Bangladesh.

## Introduction

Globally, maternal mortality ratio (MMR) has decreased by 34%, from 342 deaths per 100,000 live births in 2000 to 223 deaths per 100,000 live births in 2020 [1], and neonatal mortality rate (NMR) has halved, from 37 deaths per 1,000 live births in 1990 to 18 deaths per 1,000 live births in 2021 [2]. Despite these declining trends, about 800 women and 7,700 newborns are dying worldwide per day due to preventable causes related to pregnancy, delivery, and postpartum complications [3]. A number of medical interventions known as "signal functions" for emergency obstetric and newborn care (EmONC), defined by the United Nations (UN), are effective in treating obstetric complications as well as preventing maternal and neonatal mortality and morbidities [4,5]. Providing high-quality EmONC can reduce global maternal mortality by up to 60% and neonatal mortality by 85% annually [6,7]. EmONC interventions are categorized into basic EmONC (BEmONC) or comprehensive EmONC (CEmONC) levels, serving as benchmarks to evaluate the depth of care available. The BEmONC services comprise seven signal functions: parenteral administration of antibiotics, oxytocin, and anticonvulsants; assisted vaginal delivery; manual removal of the placenta; removal of retained products of conception; and neonatal resuscitation [8]. In comparison, the CEmONC services include two additional signal functions: blood transfusion and cesarean section [9].

Over the last few decades, Bangladesh made significant progress in reducing MMR by 60%, from 434 maternal deaths per 100,000 live births in 2000 to 173 in 2017 [1]. Similarly, NMR was also reduced by 55%, from 37 deaths per 1,000 live births in 1990 to 17 in 2020 [2]. During these periods, Bangladesh increased the coverage of essential maternal and child health care services also. However, this progress was uneven across both services and interventions. Consequently, the SDG targets of MMR less than 70 per 100,000 live births and NMR of 12 per 1000 live births have not been reached [10]. Furthermore, inadequate accessibility and availability of basic emergency obstetric and newborn care (BEmONC) [11] was one of the largest causes of maternal and neonatal mortality in Bangladesh during 2022, with rates of 156 per 100,000 live births and 17 per 1,000 live births, respectively [10]. Though Bangladesh has made significant progress in reducing MMR and NMR, the existing rates show that Bangladesh is falling behind in achieving universal coverage of basic maternal and child health services [12]. This scenario is also reflected in sub-national regions of Bangladesh, where geographical and regional disparities remain a significant challenge in achieving universal coverage of these services [13]. Further investigation is required to understand gaps in the availability and readiness of BEmONC services and any progress made in these areas, to improve future resource allocation and policy planning.

BEmONC is a cost-effective, life-saving intervention that has proven impact in reducing maternal and neonatal mortality in Bangladesh [14–16]. The UN suggests a minimum of five Basic Emergency Obstetric and Newborn Care (BEmONC) facilities for every 500,000 people [16,17]. However, a sufficient number of facilities does not guarantee service availability or readiness [5] and little is known about these elements of existing BEmONC services in Bangladesh. Identifying progress and predictors of BEmONC services is essential to designing and delivering effective interventions. Thus, we aimed to examine any changes in the availability and readiness of BEmONC services in recent decades and explore potential predictors of these services in Bangladesh.

## Materials and methods

### Study setting and data source

Bangladesh is a country in Asia that falls into the lower-middle income group. It has a population of 173 million people, making it the eighth most densely populated country in the world, with 1328 people per square kilometre [18]. The Ministry of Health and Family Welfare (MoHFW) and the Ministry of Local Government, Rural Development, and Cooperatives (MOLGRDC) are the designated ministries in Bangladesh responsible for delivering healthcare services in both rural and urban areas [19]. The health system in Bangladesh operates as a pluralistic structure with four primary actors shaping its framework: the government, private sector, non-governmental organizations (NGOs), and donor agencies. The government, as the foremost actor, holds constitutional responsibility not only for policy-making and regulation but also for providing comprehensive health services, including funding and employing healthcare personnel. The Ministry of Health and Family Welfare oversees this responsibility through two key entities: Directorates General of Health Services (DGHS) and Directorates General of Family Planning (DGFP). These bodies manage a dual system offering general health and family planning services through various healthcare facilities. These include district hospitals, Upazila Health Complexes (which typically have 10 to 50 beds) at the sub-district level, Union Health and Family Welfare Centres at the union level, and community clinics at the ward level [20].

We analysed nationally representative data from Bangladesh Health Facility Survey (BHFS) conducted nationwide in 2014 and 2017 [21,22]. The BHFS assesses the availability and readiness of essential and basic health services in health facilities across Bangladesh, including the components of BEmONC [21,22]. The National Institute of Population Research and Training (NIPORT) conducted the BHFS surveys and the data were collected by Associates for Community and Population Research (ACPR). The 2014 and 2017 BHFS collected data using standardized questions from United States Agency for International Development’s (USAID’s) Demographic and Health Surveys (DHS) program service provision assessment component. Technical assistance was provided by ICF and icddr,b [21,22].

### Sample and sampling procedure

**Fig 1:**
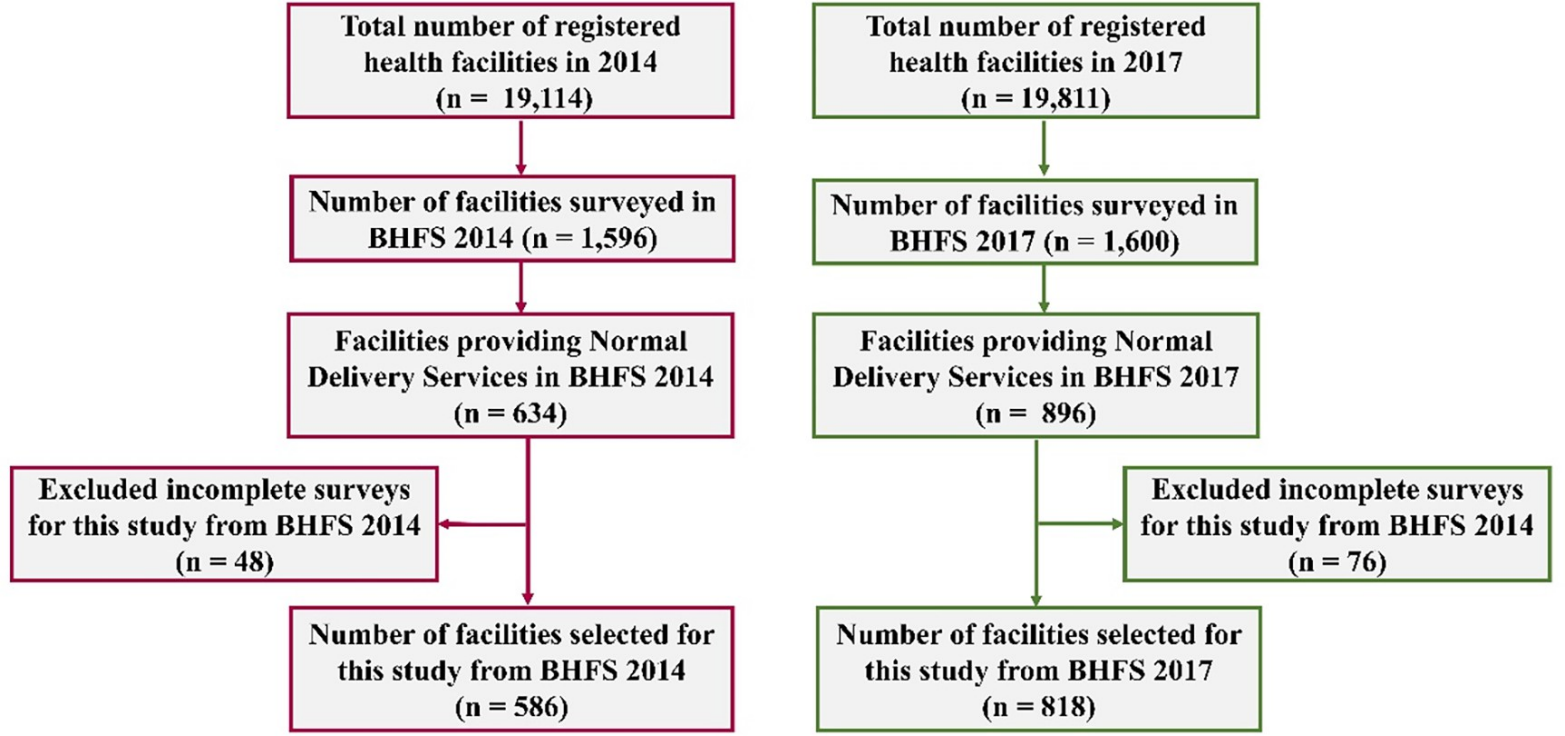
Flowchart showing the selection procedure of the sample from the BHFS 2014 and BHFS 2017.

The BHFS sampled a total of 1596 and 1600 health facilities in 2014 and 2017 respectively, using a stratified random sampling technique [21,22]. After excluding 48 and 76 health facilities due to incomplete surveys, a total of 1548 and 1524 health facilities were selected in 2014 and 2017 respectively. For the purpose of this study, the health facilities that provide normal delivery services were included in the analysis (n = 586 in 2014 and n = 818 in 2017). The selection of the sample is illustrated in **Fig 1**.

### Data collection

The BHFS 2014 and 2017 data were collected using two different types of survey instruments: a facility inventory questionnaire and a health care provider interview questionnaire [21,22]. We considered almost all variables from the facility inventory questionnaire (*Delivery and Newborn Care Section)* and one variable on staff training from the health care provider questionnaire for this study. Forty trained data collector teams were employed for data collection. The BHFS 2014 data were collected during May–July and the BHFS 2017 during July–October. A detailed description of the BHFS sampling and data collection procedure is described elsewhere [21,22].

### Measurement of variables

#### Outcome variables

**Fig 2:**
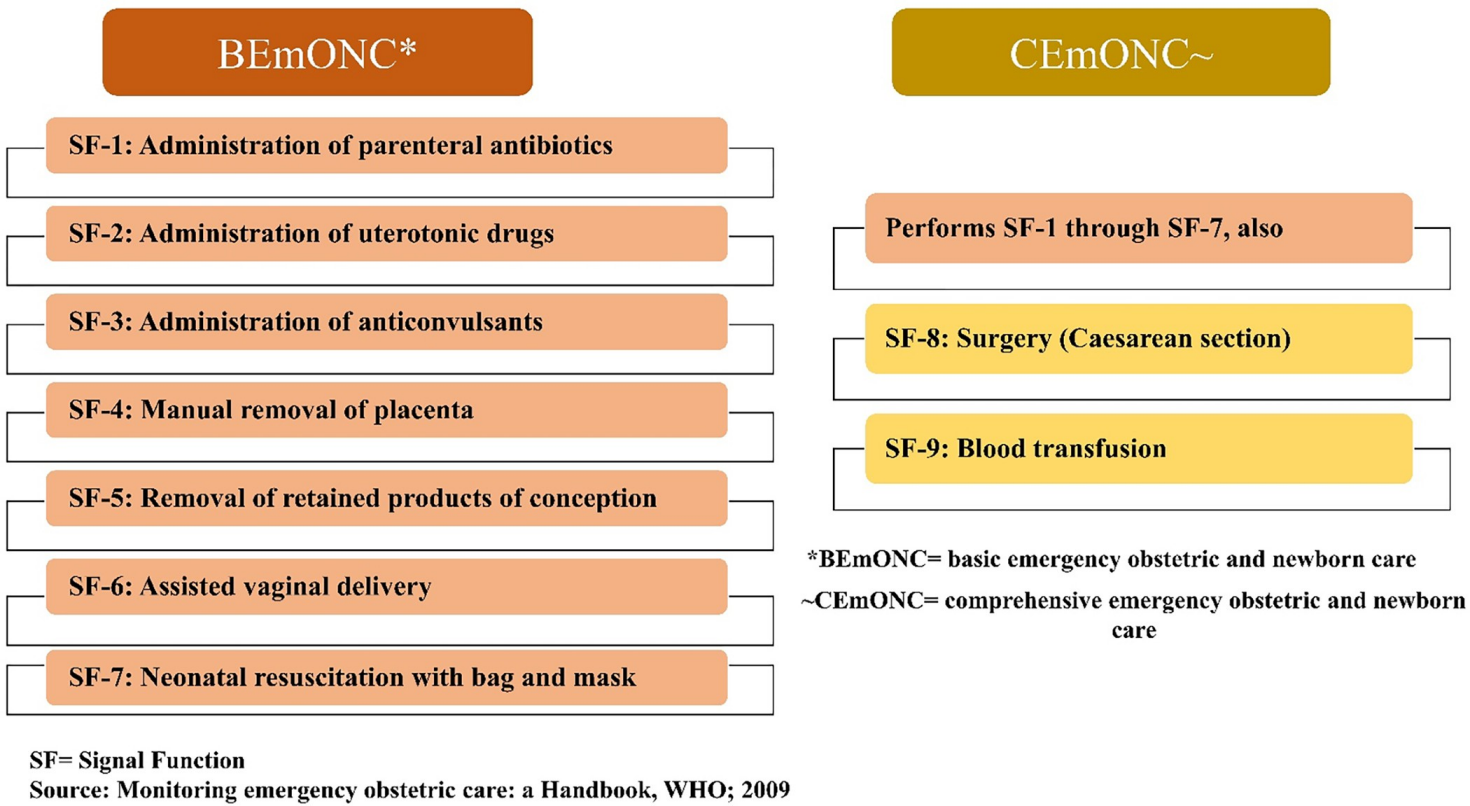
Signal functions of basic emergency obstetric care services.

The outcome variables of this study were “BEmONC service availability” and “BEmONC service readiness”. These outcome variables were selected according to the World Health Organization (WHO) service availability and readiness (SARA) guideline by the three domains of tracer indicator, each of which consists of a collection of tracer items [23]. The physical presence of services relative to the provision of BEmONC services was characterized as ‘BEmONC services availability’. BEmONC services availability was assessed using the seven signal functions of delivery and newborn care (**Fig 2**) provided by the health facility in the three months prior to the survey [17]. The respondents answering on behalf of the health facility were asked if the signal functions have been provided at least once in the previous three months. A seven-point scoring system was employed to measure BEmONC availability of designated health facilities. The availability of each signal function was given the value of one, while the absence of each signal function was given a score of zero. The BEmONC service availability score ranges from 0–7.

**Fig 3:**
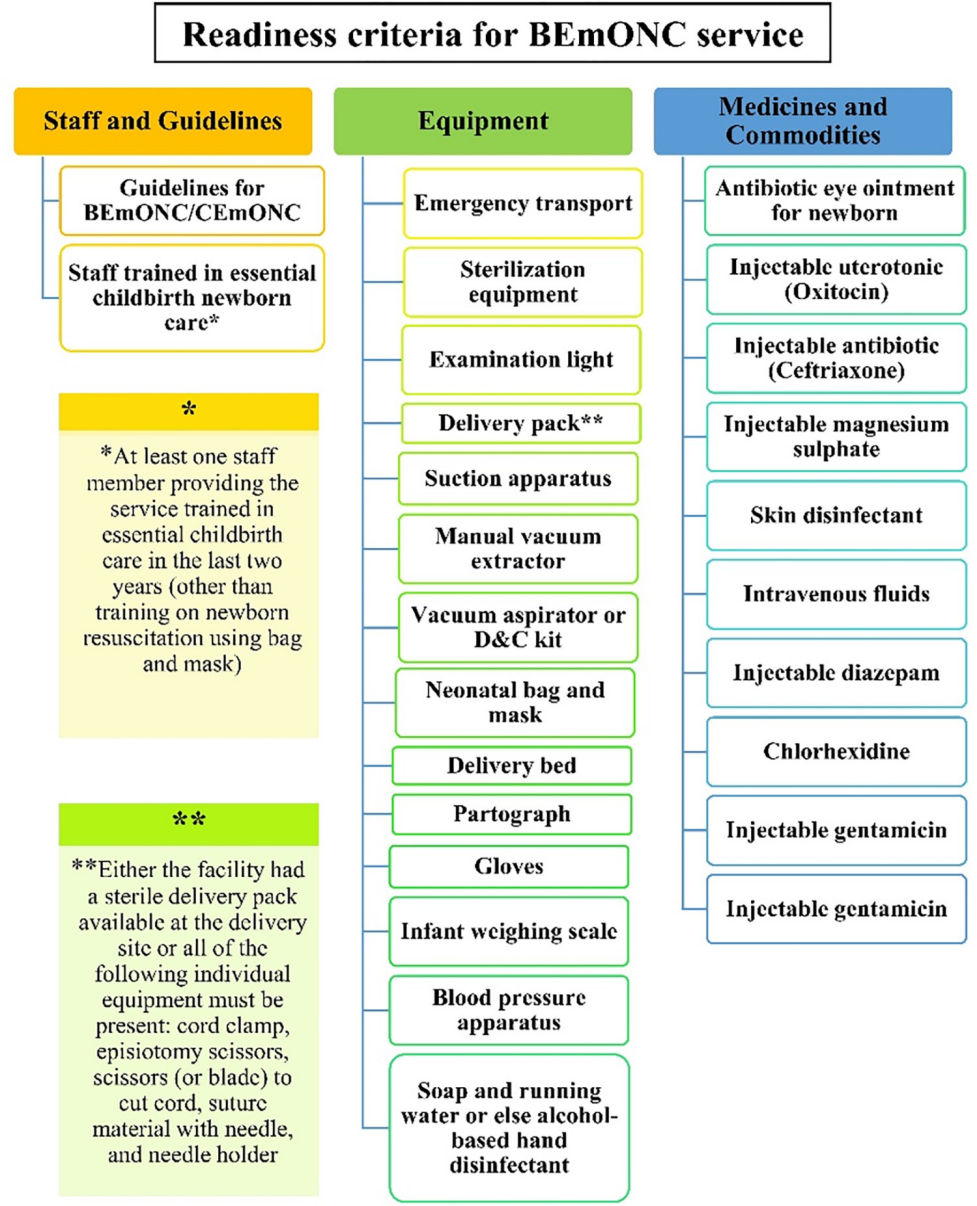
Item required for basic emergency obstetric care services.

Similarly, we defined ’BEmONC service readiness’ as the ability of the health facility to deliver BEmONC services. BEmONC service readiness was estimated based on the availability and functionality of three types of assistive objects (domains): staff and guidelines (2 indicators), basic medicine and commodities (10 indicators) and essential equipment and supplies (14 indicators) (**Fig 3**). The BEmONC service readiness was measured from the three domains considering a weighted additive approach [16]. This approach entails distributing the same weight to each domain. For the BEmONC readiness score, the total score for all three domains was standardized to a maximum of 100 points [16]. Under the weighted additive approach, the three domains were initially weighted equally at 33.33%. Subsequently, the domain scores were allocated based on the number of indicators within each domain. For instance, the Equipment domain comprised 14 indicators, leading to the division of the 33.33% score for the domain by 14, resulting in an assignment of 2.38 to each indicator within this domain. The detailed procedure for calculating readiness score are available in the **S1 Table in S1 File**.

#### Independent variables

We selected the independent variables based on existing literature [16,24] and availability of information in both surveys. The selected independent variables include: location of the facility (Rural or Urban), division of the facility (Dhaka, Chattogram, Barishal, Khulna, Rajshahi, Rangpur, Mymensingh, Sylhet), duty schedule for 24-hour staff assignment (Yes and No), opinion giving option for client (Yes and No), reviews of maternal or newborn deaths (Yes and No), quality assurance performed at least once a year (performed and not performed), types of health facility types (District Hospital (DH), Maternal and Child Welfare Center (MCWC), Upazila Health Complex (UHC), Upazila Health and Family Welfare Center (UH&FWC), Union Subcenter/ Rural Dispensary (UnSC/RD), Community Clinic (CC), Private and NGO Clinic/Hospitals). We also considered survey year in the regression analysis to control the variations due to time.

### Statistical analysis

We performed univariate analysis to calculate the weighted mean for continuous variables and percentage for categorical variables. The mean was reported along with standard deviation (SD) and percentage with 95% confidence intervals (CIs). The number of each type of facility were weighted so that their contribution to the total became proportional to the country’s actual distribution of health facilities [22]. We considered facility weights to restore the representativeness of the health facilities sampled. As BEmONC service availability denotes the number of services available, we applied a robust Poisson regression model to assess the factors associated with BEmONC services availability.

The results of the Poisson regression model with robust variance are presented in terms of prevalence ratio along with 95% confidence intervals (CIs). Also, we fitted linear regression models to determine the factors associated with the continuous BEmONC services readiness scores. The results of the linear regression analysis are presented in terms of adjusted coefficients along with 95% CIs. We calculated the variance inflation factor (VIF) to examine whether the independent variables were correlated and found absence of multicollinearity. Stata’s “svyset” commands were used to account for the BHFS complex sample design (sample weight and cluster) for estimating standard errors (SEs). A 5% level of significance has been used to define statistical significance for all two-sided tests performed. Data were analysed in Stata version 16.0 (Stata Statistical Software, College Station, Texas, USA). Additionally, we utilized ArcGIS version 10.8 to prepare the map. We accessed the District Shapefile of Bangladesh from the Bangladesh Agricultural Research Council (BARC) through registration on its website to obtain the map layout.

Ethics approval and consent to participate. The survey analysed was approved by the Institutional Review Board of ICF International. Administrative approval was obtained from the National Research Ethics Committee of the Bangladesh Medical Research Council and MoH&FW before data collection. We obtained permission to access this survey and conduct this research from the Demographic and Health Survey’s Program following standard protocol. No additional ethical approval was required to conduct this study.

## Results

### Distribution of health facilities

Analysis included 280 facilities that provided normal delivery in 2014 and 358 in 2017 BHFS. The majority of the facilities that provided normal delivery services were located in rural areas (70.7% vs 29.3% in urban in 2014 and 78.3% vs 21.7% in urban in 2017). The number of facilities providing normal delivery declined in Sylhet, Rangpur, Dhaka and Chittagong whilst increasing or remaining unchanged in other divisions. Normal delivery services were largely provided in the UH&FWCs in both 2014 (32.7%) and 2017 (45.2%), and these facilities showed the largest increase in provision of normal delivery services. In contrast, DHs, MCWCs, UHCs, CCs, Private and NGO clinic/hospitals showed a decline in the percentage of facilities providing normal delivery services between 2014 and 2017 (**Table 1**).

**Table 1:** Percentage distribution (Weighted) of surveyed facilities which provide normal delivery services, BHFS 2014 and 2017.

Variable	n (%)	
	2014	2017
**Location of facility**		
Urban	82 (29.3)	78 (21.7)
Rural	198 (70.7)	280 (78.3)
**Division**		
Barishal	15 (5.4)	33 (9.1)
Chattogram	58 (20.5)	80 (9.1)
Dhaka	97 (34.6)	78 (21.9)
Khulna	25 (8.9)	36 (10.0)
Mymensingh	-	21 (6.0)
Rajshahi	35 (12.58)	54 (15.2)
Rangpur	33 (11.8)	38 (11.0)
Sylhet	18 (6.3)	19 (5.2)
**Health Facility Type**		
DH	5 (1.8)	5 (1.3)
MCWC	7 (2.5)	6 (1.8)
UHC	34 (12.0)	31 (8.6)
UH &FWC	92 (32.7)	162 (45.2)
UnSC/RD	12 (4.2)	28 (7.7)
CC	73 (26.1)	66 (18.4)
Private	33 (11.9)	41 (11.3)
NGO clinic/hospital	25 (8.9)	20 (5.7)
**Total**	**280 (100)**	**358 (100)**

### Availability of BEmONC services

**Fig 4:**
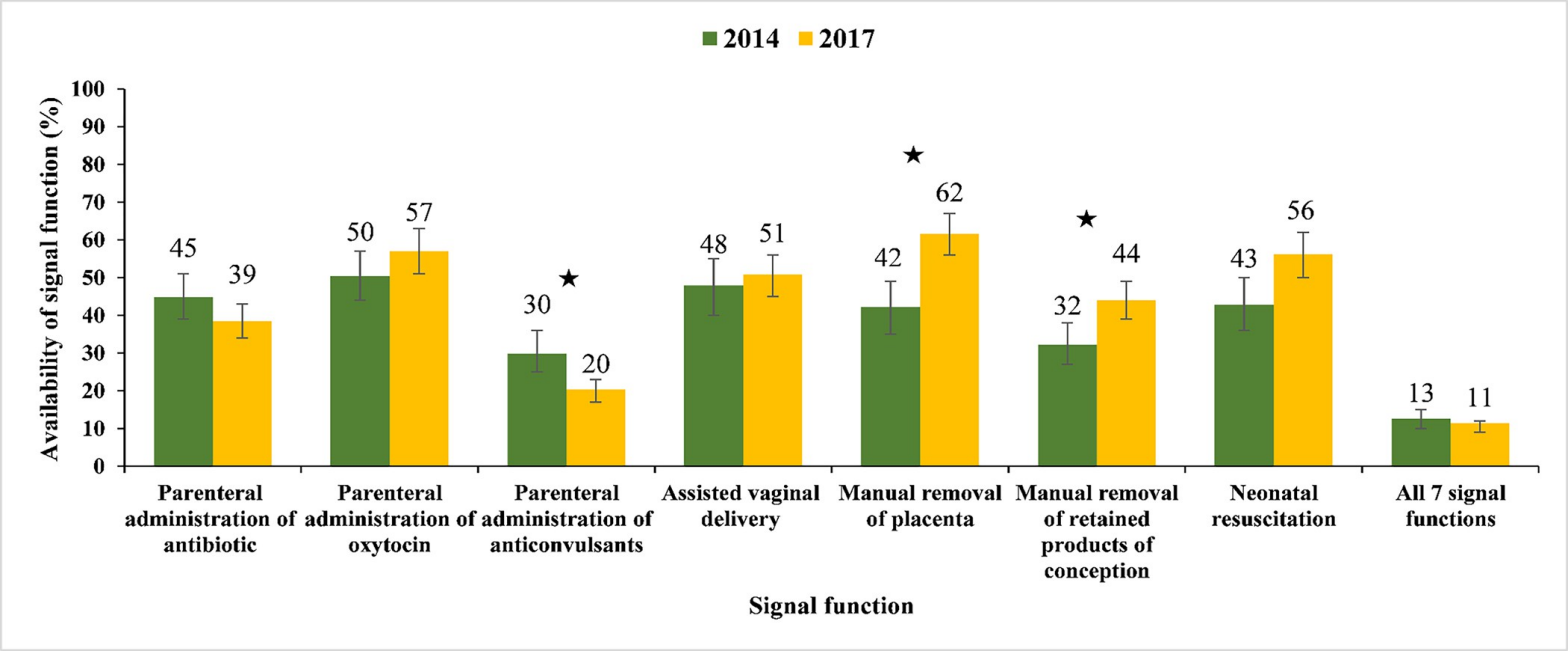
Availability of BEmONC services in health facilities providing normal delivery in Bangladesh, 2014 and 2017. Asterisks (*) represent statistical significance (denotes p-value<0.05).

**Fig 4** depicts that the percentage of health facilities with availability of all seven signal functions for BEmONC declined from 13% in 2014 to 11% in 2017. However, this decline was not statistically significant. Item-specific estimates of signal functions reveals that the percentage of facilities with manual removal of placenta increased from 42% in 2014 to 62% in 2017, and facilities with manual removal of retained products of conception increased from 32% in 2014 to 44% in 2017. Conversely, the availability of facilities with parenteral administration of anticonvulsants decreased from 30% in 2014 to 20% in 2017. The percentage of facilities with availability of other services remain unchanged between 2014 and 2017 (**Fig 4**).

The availability of all seven signal functions decreased in UHCs (36.3% to 25.1%), UnSC/RDs (7.9% to 0.4%), and NGO clinics/hospitals (27.6% to 9.0%) from 2014 to 2017. The availability of most signal functions increased in DHs, CCs, NGO clinics/hospitals, and private health facilities, and decreased in MCWCs, UHCs, and UnSC/RDs from 2014 to 2017. The availability of neonatal resuscitation increased in all types of facilities from 2014 to 2017. The percentages of facilities where all seven signal functions were available decreased in all divisions from 2014 to 2017, except Rangpur (**S2 Table in S1 File**).

**Fig 5:**
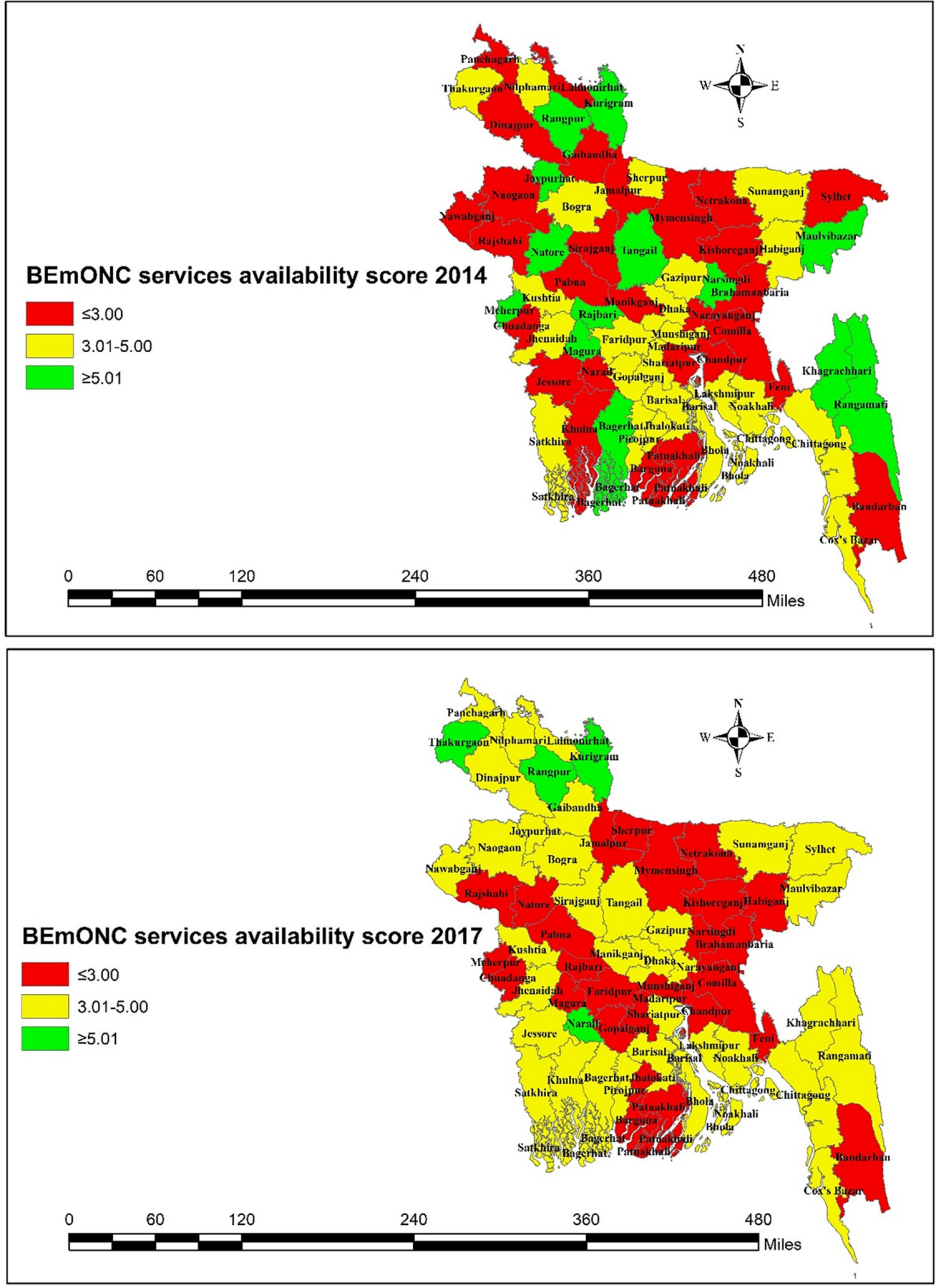
Facilities with availability of basic emergency obstetric care services across districts in Bangladesh.

**Fig 5** shows temporal variations in facilities with the BEmONC service availability score across 64 districts in Bangladesh. In 2014, 28 districts had facilities with services available for three or less signal functions, reduced to 26 districts in 2017. The number of districts that had facilities with at least five services available also declined from 13 districts in 2014 to 4 districts in 2017. This shift was largely observed in the Eastern, Western, and Southern districts in Bangladesh.

The results of the robust Poisson regression analysis are summarized in **Table 2.** Health facilities in Chattogram and Rangpur divisions had 1.18 (CI: 1.05–1.33) and 1.28 (CI: 1.10–1.48) times higher rates respectively of available BEmONC services compared to the health facilities in Dhaka division, given the other variables are held constant in the model. The facilities where maternal and newborn deaths were reviewed had a 1.22 (CI: 1.11–1.35) times higher rate of having BEmONC services available than their counterpart facilities where these deaths were not reviewed, after controlling for other covariates. The UH&FWCs, UnSC/RDs, and CCs had 0.5 (CI: 0.41–0.60), 0.61 (CI: 0.43–0.85) and 0.36 (CI: 0.17–0.76) times lower rates respectively of BEmONC service availability compared to DHs, after adjusting for other variables.

**Table 2:** Factors associated with the availability of basic emergency obstetric care services.

Variable	Prevalence ratio (95% confidence interval)	
	Unadjusted	Adjusted
**Location of facility**		
Rural	Reference	Reference
Urban	2.14 (1,92, 2.38) ***	1.04 (0.93, 1.17)
**Division**		
Dhaka	Reference	Reference
Barisal	0.97 (0.76, 1.23)	1.2 (1.06, 1.37) **
Chattogram	1.09 (0.88, 1.34)	1.18 (1.05, 1.33) **
Khulna	1.07 (0.87, 1.32)	1.13 (1.00, 1.29)
Mymensingh	0.76 (0.58, 1.01)	0.87 (0.71, 1.08)
Rajshahi	1.00 (0.77, 1.31)	1.13 (0.97, 1.33)
Rangpur	1.39 (1.13, 1.73) **	1.28 (1.10, 1.48) **
Sylhet	0.97 (0.74, 1.28)	1.05 (0.91, 1.22)
**Duty schedule for 24 hours**		
No	Reference	Reference
Yes	1.94 (1.66, 2.27) ***	1.1 (0.95, 1.26)
**Opinion giving option for client**		
No	Reference	Reference
Yes	1.58 (1.38, 1.81) ***	1.11 (1.00, 1.23)
**Review maternal/newborn death**		
No	Reference	Reference
Yes	1.85 (1.64, 2.09) ***	1.22 (1.11, 1.35) ***
**Health Facility Type**		
DH	Reference	Reference
MCWC	0.79 (0.74, 0.84) ***	0.9 (0.83, 0.98) *
UHC	0.83 (0.78, 0.88) ***	0.89 (0.82, 0.96) **
UH &FWC	0.36 (0.33, 0.40) ***	0.5 (0.41, 0.60) ***
UnSC/RD	0.42 (0.34, 0.51) ***	0.61 (0.43, 0.85) **
CC	0.32 (0.23, 0.45) ***	0.36 (0.17, 0.76) **
NGO clinic/hospital	0.67 (0.57, 0.78) ***	0.79 (0.68, 0.93) **
Private	0.89 (0.84, 0.95) ***	1.01 (0.94, 1.08)
**Survey year**		
2014	Reference	Reference
2017	1.31 (0.98, 1.30)	1.08 (0.99, 1.17)

Note

*** denotes p-value<0.001

** denotes p-value<0.01

* denotes p-value<0.05.

### Readiness of BEmONC services

**Fig 6:**
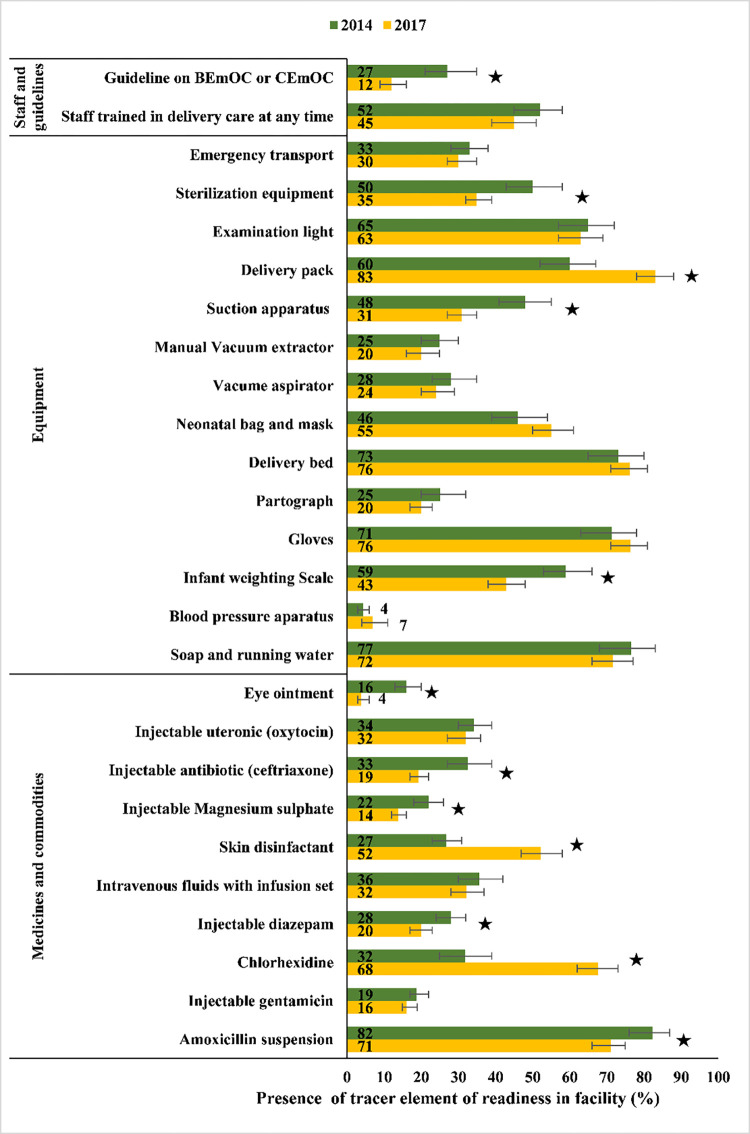
Tracer element readiness percentages in health facilities providing normal delivery in Bangladesh, 2014 to 2017. Asterisks (*) represent statistical significance (denotes p-value<0.05).

**Fig 7:**
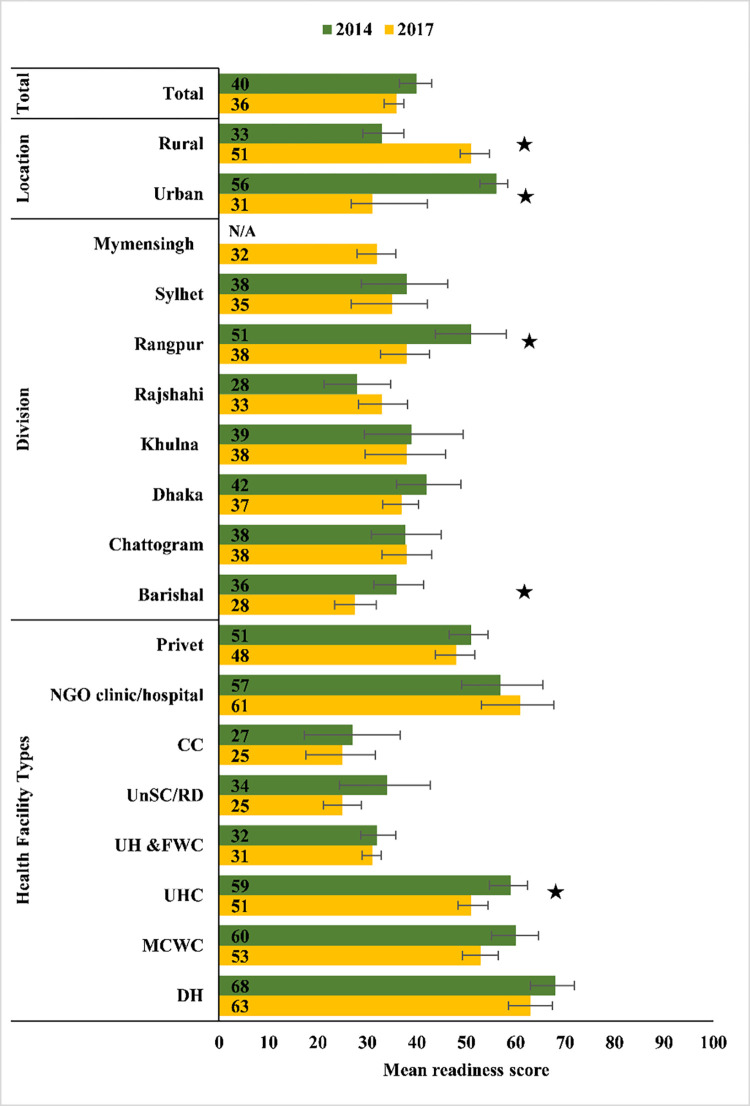
Mean readiness score for providing BEmONC services according to location, division and type of facility. Asterisks (*) represent statistical significance (denotes p-value<0.05).

BEmONC service readiness is depicted in **Fig 6** across three domains: staff and guidelines, equipment and supplies, and medicines and commodities. The percentage of health facilities having recommended guidelines related to BEmONC/CEmONC decreased significantly from 27% in 2014 to 12% in 2017. Compared to 2014, facilities with suction apparatus decreased significantly by 17%, from 48% to 31% in 2017. Similarly, facilities with infant weighting scales decreased significantly from 59% in 2014 to 43% in 2017. In the medicines and commodities domain, a significant increased number of facilities reported the presence of chlorhexidine (up from 32% in 2014 to 68% in 2017) and skin disinfectant (up from 27% in 2014 to 52% in 2017). However, significantly fewer facilities had eye ointment, injectable antibiotic, injectable magnesium sulphate, injectable diazepam and amoxicillin suspension in 2017 compared to 2014 (**Fig 6**). Apart from Barishal division, the mean health facility readiness score did not change significantly overall, or across location, division or type of health facility between 2014 and 2017. Barishal division showed a significant decline in mean readiness score from 36 in 2014 to 28 in 2017 (**Fig 7**).

**Fig 8:**
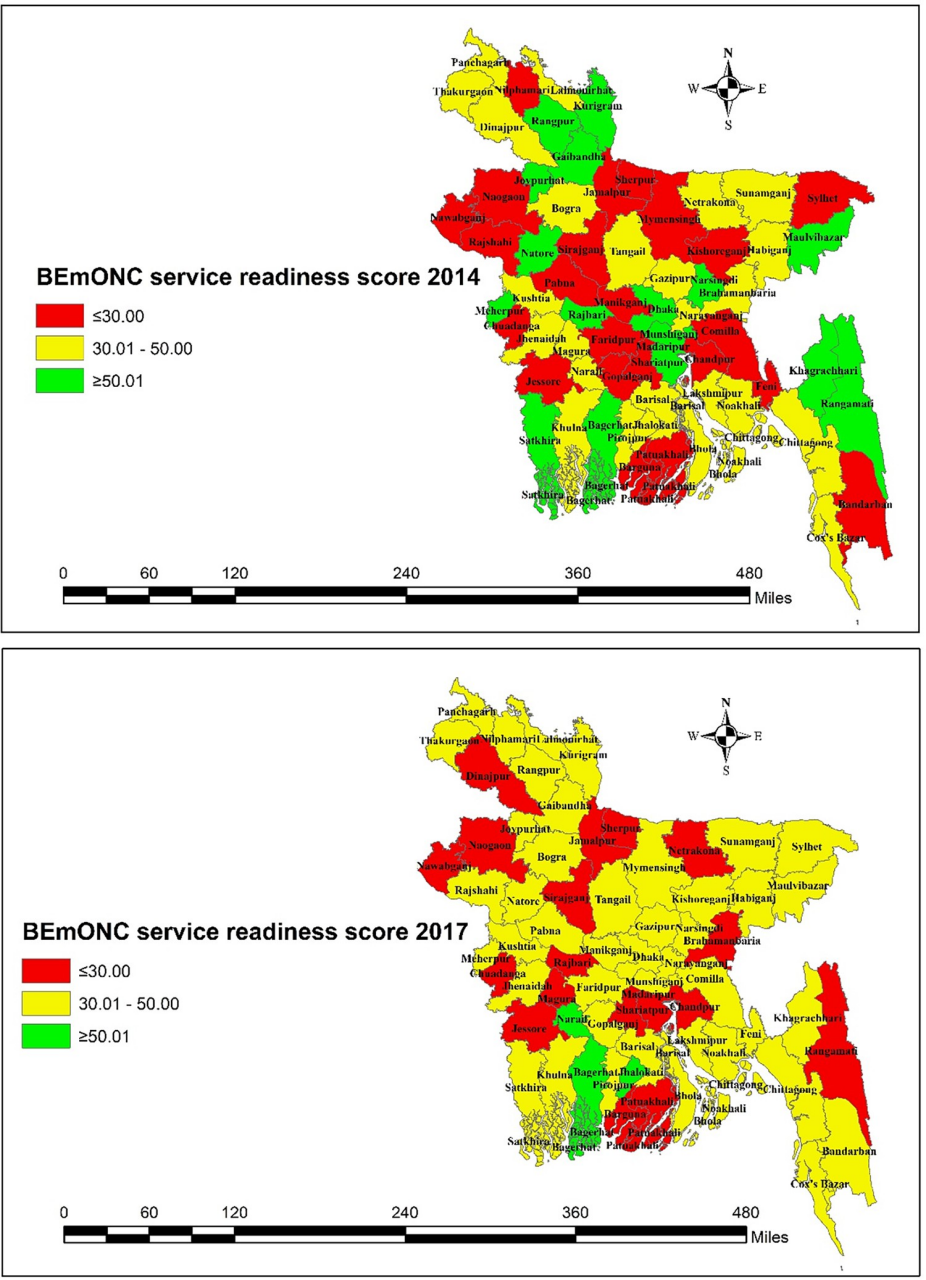
BEmONC service readiness in health facilities by district-wise in Bangladesh.

Similarly, to the temporal variations in the availability of BEmOC services across districts, the mean readiness score of health facilities also varied across districts over time. A sharp fall in the number of health facilities with a mean readiness score of at least 50 was observed, from 16 districts in 2014 to 3 districts in 2017. Only some of the southern districts in Bangladesh retained a mean readiness score of ≥50 in both 2014 and 2017 (**Fig 8)**.

Findings from the multiple linear regression analysis presented in **Table 3** show that the BEmONC readiness score increased by an average of 5 units (adjusted coefficient: 5.40; CI: 1.05–9.74) at facilities that had 24-hour duty schedules compared to facilities that did not, after adjusting for other covariates. Facilities with the option for clients to give their opinion scored 7 units higher on average (adjusted coefficient: 7.04; CI: 3.62–10.45), demonstrating a positive impact of client engagement on BEmONC readiness, and those performing quality assurance scored 4 units higher on average (adjusted coefficient: 4.27; CI: 0.57–7.97), for BEmONC readiness compared to their counterparts, after accounting for other covariates. The average readiness score was lower in UH&FWCs, UnSC/RDs and CCs compared to DHs, by 20 units (adjusted coefficient: -20.48; CI: -32.09, -8.88), by 20 units (adjusted coefficient: -19.63; CI: -33.15, -6.11), and by 15 units (adjusted coefficient: -15.38; CI: -28.29, -2.48) respectively, after controlling for other covariates.

**Table 3:** Factors associated with the readiness of basic emergency obstetric care services.

Variable	Coefficient (95% confidence interval)	
	Unadjusted	Adjusted
**Location of facility**		
Rural	Reference	Reference
Urban	22.55 (20.67, 24.44) ***	3.45 (-1.92, 8.82)
**Division**		
Dhaka	Reference	Reference
Barishal	-6.23 (-10.08, -2.50) **	0.5 (-5.73, 6.72)
Chattogram	-0.57 (-5.00,2.95)	2.02 (-2.25, 6.29)
Khulna	2.47 (-1.98, 6.92)	2.45 (-3.69, 8.58)
Mymensingh	-9.4 (-15.42, -3.56) **	-6.15 (-14.42, 2.13)
Rajshahi	-4.22 (-8.49, 0.61)	-0.99 (-6.42, 4.43)
Rangpur	2.50 (-1.93, 6.93)	3.54 (-2.34, 9.42)
Sylhet	-3.63 (-7.96, 0.70)	0.55 (-6.51, 7.61)
**Duty schedule for 24 hours**		
No	Reference	Reference
Yes	21.26 (18.86, 23.67) ***	5.4 (1.05, 9.74) *
**Opinion giving option for client**		
No	Reference	Reference
Yes	15.07 (12.99, 17.16) ***	7.04 (3.62, 10.45) ***
**Review maternal/newborn death**		
No	Reference	Reference
Yes	15.23 (13.16, 17.31) ***	3.39 (-0.16, 6.93)
**Quality assurance**		
Not performed	Reference	Reference
Performed	16.27 (14.19,18.34) ***	4.27 (0.57, 7.97) *
**Health Facility Type**		
DH	Reference	Reference
MCWC	-11.74 (-15.60, -7.88) ***	-3.81 (-16.48, 8.86)
UHC	-9.47 (-13.0, -5.95) ***	-6.61 (-16.92, 3.70)
UH &FWC	-33.46 (-41.60, -32.23) ***	-20.48 (-32.09, -8.88) **
UnSC/RD	-36.92 (-41.61, -32.23) ***	-19.63 (-33.15, -6.11) **
CC	-41.75 (-47.39, -36.10) ***	-15.38 (-28.29, -2.48) *
Private	-9.11 (-13.26, -4.95) ***	-1.7 (-12.10, 8.69)
NGO clinic/hospital	-9.13 (-13.06, -5.20) ***	-7.24 (-17.47, 2.98)
**Survey year**		
2014	Reference	Reference
2017	-6.45 (-8.67, -4.23) ***	-0.76 (-4.16, 2.64)

Note

*** denotes p-value<0.001

** denotes p-value<0.01

* denotes p-value<0.05.

## Discussion

This study reveals a decline in the availability of all seven basic signal functions in health facilities from 2014 to 2017, predominantly driven by declines in MCMCs, UHCs and UnSC/RDs, and in all divisions except Rangpur. Regular reviewing of maternal and neonatal deaths was significantly associated with increased incidence of BEmONC service availability at health facilities. No significant changes in the overall readiness of health facilities were observed between 2014 and 2017. However, fewer facilities reported to have BEmONC guidelines, and medicines and commodities in 2017 compared to 2014 despite an increase in the presence of chlorhexidine and skin disinfectant across health facilities during this time. Facilities with 24-hour duty schedules, options for clients to provide opinions and that conducted quality assurance checks were associated with greater readiness of the health facilities in Bangladesh. To the best of our knowledge, this is the first study to use a nationally representative sample to assess changes in the availability and readiness of BEmONC services at national and subnational levels, and across different types of health facilities in Bangladesh.

The static state of overall readiness in Bangladesh’s health facilities contrasts with another study conducted in Nepal and Tanzania, where the overall availability of seven signal functions [24,25], and average readiness score significantly increased respectively [24]. The decline in the availability of signal functions and BEmONC service readiness in Bangladesh is largely driven by changes at UH&FWC, UnSC/RD and CC facilities. Despite improvements in DHs, MCWCs, and UHCs, performance was still below satisfactory levels. However, improvements in these facilities are not surprising as BEmoNC services are expected to be provided by DHs, MCWCs and UHCs [26]. According to the Bangladesh Essential Health Service Package (ESP), aiming to reduce maternal mortality in Bangladesh, all UH&FWCs and UnSCs, and some selected CCs, should also have the provision of BEmONC services and it is therefore mandatory for these facilities to provide normal delivery services [26]. Despite the WHO recommending the availability and accessibility of EmOC services to all mothers [17], specifically providing services for BEmONC signal functions are not listed as essential service for union level facilities in the Bangladesh ESP or Bangladesh National Strategy for Maternal Health [26,27]. Therefore, it is imperative to include signal functions in the list of essential service packages for facilities that provide normal delivery services in Bangladesh. Given that the Government of Bangladesh is currently developing the 5th sector program subsequent to the successful conclusion of the 4th sector program [28], it is now opportune for policymakers to address this issue.

Despite government regulations that consider conducting normal deliveries in CCs as an additional service and providing BEmONC is not its responsibility [29], the availability of signal functions for BEmONC was surprisingly good in CCs compared to other union level facilities (UH&FWC and UnSC/RDs). A 2009 government project named "Revitalisation of Community Healthcare Initiatives in Bangladesh" prioritized the strengthening of CCs and could possibly be a reason for these findings [30]. Until 2019, a total of 1,935 female Community Healthcare Providers (CHCPs) from CCs received training on conducting normal delivery [30]. Additionally, the government increased budget allocation for strengthening the supply of medicine in CCs by 32 times (from 6.5 crore in BDT to 210 crore in BDT) from 2008–09 to 2018–19 [30].

The government also considered UHCs as a strategic location and started working to modernise the BEmONC services with necessary human resources, infrastructure, and equipment [26,27]. However, this study highlighted the availability of most signal functions decreased from 2014 to 2017 in UHCs. Moreover, only one-fourth of the UHCs were providing all seven signal functions in 2017. As the first referral facility, UHCs should be ready for providing 24/7 maternity care and BEmONC services [27]. Despite such recommendations in the BESP, the reduction of signal functions in UHCs is alarming, and requires special attention. The lack of monitoring and programmatic investment in these facilities can potentially explain the reduction in availability of signal functions.

Our analysis reveals low availability of the seven signal functions in rural health facilities in Bangladesh in both survey years. The services availability of two signal functions in particular (parenteral administration of antibiotic and anticonvulsants) reduced in rural facilities, while the availability of the seven signal functions increased in urban health facilities. This highlights a substantial gap regarding the BEmONC service readiness in rural health facilities compared to urban health facilities, consistent with findings from other countries, including Nepal [16], Kenya [31], and Madagascar [32]. Usually all referral level facilities are located in urban areas where BEmONC should be provided [29] and may be a reason for this finding. Lower availability and readiness of facilities for BEmONC services in rural health facilities is a significant barrier to effective BEmONC services for all rural women [16]. The trends in BEmONC service availability from 2014 to 2017 revealed alarmingly inadequate maternal and neonatal healthcare even before the health systems faced additional strain from the COVID-19 pandemic. Bangladesh initially struggled during the COVID-19 period but managed a strong recovery [33]. Though Bangladesh has made significant progress in several maternal and neonatal healthcare service indicators in recent years even after COVID-19 pandemic [34], the impact of the COVID-19 shock may continue to affect the availability of BEmONC services.

Around 80% of the surveyed health facilities did not have a trained staff member in delivery care wards in both years. This raises critical concerns about the quality of normal delivery services, including BEmONC. Additionally, in 2017, more than half of health facilities did not have any emergency transport, sterilization equipment, suction apparatus, manual vacuum extractors, vacuum aspirators, partographs and blood pressure apparatus. This exposes the gaps in national program planning and implementation, warranting urgent action for increasing the availability and readiness of BEmONC services.

In this study, the availability of signal functions was significantly associated with conduction of periodic reviews of maternal and newborn deaths, and readiness scores were significantly associated with both provision of 24-hour duty schedules and opinion giving options for clients. This shows that health institutions’ infrastructure, review processes, and human resource aspects need to be improved in order to improve overall BEmONC service availability and readiness [16]. The government of Bangladesh has already started the maternal perinatal death surveillance and response (MPDSR) to find out where, when, how, and why the mother or newborn died, and aims to use this information to prevent future deaths [35].

### Strength and limitation

The present study has several strengths. First and foremost, to our knowledge, this exercise is the first of its kind to examine the progress towards the availability and readiness of BEmONC services utilising nationally representative data in Bangladesh. The BHFS 2014 and 2017 used a nationally representative sample, allowing us to generate estimates based on location, facility type, and division. Additionally, this study utilised the globally accepted SARA guidelines to estimate the availability and readiness of BEmONC services. Further strength of this study was the data collection tool utilised in both survey years was verified and modified to the national context via expert consultations.

There were some limitations to this excercise also. The dataset utilised in this study spans the period between 2014 and 2017, rendering it more than six years old. Additionally, because the survey assessed the availability of services and items on the day of the visit, this only provides a cross-sectional snapshot of the service availability and readiness of the studied facilities. Besides, the information on service availability is based on the facility’s self-reported status whereas readiness was assessed through physical observations by data collectors to confirm the availability of necessary and essential items, as well as their functionality, where appropriate. Self-reported response is common in many studies and is useful to understand many issues of interest. Lastly, it is important to acknowledge that disparities in service availability and readiness may be the consequence of bigger health system building block challenges, such as health funding and governance. We were unable to investigate and provide root causes due to a lack of data, which need to be investigated further in future studies.

## Conclusion

Our study findings demonstrate the reduction of BEmONC service availability and readiness in 2017 compared to 2014 in Bangladesh. The observed reductions varied substantially across divisions and districts. Parenteral administration of anticonvulsants was the least available signal function in the facilities in both 2014 and 2017 survey years. The availability and readiness of BEmONC services was lowest in union level facilities. The availability of most signal functions decreased in UHCs and increased in CCs over the study period. The results of this study are expected to be useful in identifying areas where maternal and neonatal health care services require improvement in order to reduce maternal and neonatal mortality and morbidity. Bangladesh should make significant efforts to strengthen program design, implementation, and monitoring as an attempt to reduce these crucial gaps and provide better care for mothers and children during normal delivery. Moreover, research on improving delivery strategies and addressing challenges to BEmONC service implementation could accelerate progress in increasing the availability and readiness of health facilities for BEmONC services, and therefore, facilitate to achieve SDGs regarding MMR and NMR by 2030.

## Supporting information

S1 File (DOCX)

## Abbreviations

BEmONC: Basic Emergency Obstetric and Newborn Care
BHFS: Bangladesh Health Facility Survey
CC: Community Clinic
CEmONC: Comprehensive Emergency Obstetric and Newborn Care
Cis: confidence intervals
DH: District Hospital
DHS: Demographic and Health Surveys
EmONC: Emergency Obstetric and Newborn Care
MCWC: Maternal and Child Welfare Center
MMR: maternal mortality ratio
NIPORT: National Institute of Population Research and Training
NMR: neonatal mortality rate
UH&FWC: Upazila Health and Family Welfare Center
UHC: Upazila Health Complex
UnSC/RD: Union Subcenter/ Rural Dispensary
WHO: World Health Organization
