# Supplementary material for: Measuring progress in availability and readiness of Basic emergency obstetric and newborn care (BEmONC) services in Bangladesh, 2014–2017
Source: PLoS One. 2025 Feb 14;20(2):e0314116. doi: 10.1371/journal.pone.0314116 (PMC11828372; doi:10.1371/journal.pone.0314116)
Supplement: S1 File — (DOCX) [file pone.0314116.s001.docx]

**Supplementary Table 1. Procedure for calculating the readiness score**

| **Domain** | **Indicators (Tracer items)** | **Measurement** | **Percent score (%)** | |
| --- | --- | --- | --- | --- |
|  |  |  | **Indicator** | **Domain** |
| **Delivery and new born care index** | | | | |
| Staff and guidelines (2 indicators) | Guidelines for BEmONC/CEmOC | Yes | 16.67 | 33.33 |
|  |  | No | 0.00 |  |
|  | Staff trained in essential childbirth newborn care**^1^** | Yes | 16.67 |  |
|  |  | No | 0.00 |  |
| Equipment (14 Indicator) | Emergency transport | Yes | 2.38 | 33.33 |
|  |  | No | 0.00 |  |
|  | Sterilization equipment | Yes | 2.38 |  |
|  |  | No | 0.00 |  |
|  | Examination light | Yes | 2.38 |  |
|  |  | No | 0.00 |  |
|  | Delivery pack^2^ | Yes | 2.38 |  |
|  |  | No | 0.00 |  |
|  | Suction apparatus (mucus extractor) | Yes | 2.38 |  |
|  |  | No | 0.00 |  |
|  | Manual vacuum extractor | Yes | 2.38 |  |
|  |  | No | 0.00 |  |
|  | Vacuum aspirator or D&C kit (with speculum) | Yes | 2.38 |  |
|  |  | No | 0.00 |  |
|  | Neonatal bag and mask | Yes | 2.38 |  |
|  |  | No | 0.00 |  |
|  | Delivery bed | Yes | 2.38 |  |
|  |  | No | 0.00 |  |
|  | Partograph | Yes | 2.38 |  |
|  |  | No | 0.00 |  |
|  | Gloves | Yes | 2.38 |  |
|  |  | No | 0.00 |  |
|  | Infant weighing scale | Yes | 2.38 |  |
|  |  | No | 0.00 |  |
|  | Blood pressure apparatus | Yes | 2.38 |  |
|  |  | No | 0.00 |  |
|  | Soap and running water or else alcohol-based hand disinfectant | Yes | 2.38 |  |
|  |  | No | 0.00 |  |
| Medicines and commodities  (10 Indicator) | Antibiotic eye ointment for newborn | Yes | 3.33 | 33.33 |
|  |  | No | 0.00 |  |
|  | Injectable uterotonic (Oxitocin) | Yes | 3.33 |  |
|  |  | No | 0.00 |  |
|  | Injectable antibiotic (Ceftriaxone) | Yes | 3.33 |  |
|  |  | No | 0.00 |  |
|  | Injectable magnesium sulphate | Yes | 3.33 |  |
|  |  | No | 0.00 |  |
|  | Skin disinfectant | Yes | 3.33 |  |
|  |  | No | 0.00 |  |
|  | Intravenous fluids | Yes | 3.33 |  |
|  |  | No | 0.00 |  |
|  | Injectable diazepam | Yes | 3.33 |  |
|  |  | No | 0.00 |  |
|  | Chlorhexidine | Yes | 3.33 |  |
|  |  | No | 0.00 |  |
|  | Injectable gentamicin | Yes | 3.33 |  |
|  |  | No | 0.00 |  |
|  | Amoxicillin suspension | Yes | 3.33 |  |
|  |  | No | 0.00 |  |
| **Total Delivery and newborn care index score** | | | | 100 |
| ^1^At least one staff member providing the service trained in essential childbirth care in the last two years (other than training on newborn resuscitation using bag and mask) | | | | |
| ^2^Either the facility had a sterile delivery pack available at the delivery site or all of the following individual equipment must be present: cord clamp, episiotomy scissors, scissors (or blade) to cut cord, suture material with needle, and needle holder | | | | |

**Supplementary Table 2. Percentage distribution of seven signal functions for basic emergency obstetric and newborn care services in Bangladesh**

|  | **Parenteral administration of antibiotic** | | **Parenteral administration of oxytocin** | | **Parenteral administration of anticonvulsants** | | **Assisted vaginal delivery** | | **Manual removal of placenta** | | **Manual removal of retained products of conception** | | **Neonatal resuscitation** | | **3 signal functions^1^** | | **All 7 signal functions** | |
| --- | --- | --- | --- | --- | --- | --- | --- | --- | --- | --- | --- | --- | --- | --- | --- | --- | --- | --- |
|  | **2014** | **2017** | **2014** | **2017** | **2014** | **2017** | **2014** | **2017** | **2014** | **2017** | **2014** | **2017** | **2014** | **2017** | **2014** | **2017** | **2014** | **2017** |
| **Health Facility Type** |  |  |  |  |  |  |  |  |  |  |  |  |  |  |  |  |  |  |
| DH | 95.1 | 96.8 | 90.2 | 95.2 | 78.7 | 88.7 | 85.2 | 75.8 | 93.4 | 96.8 | 80.3 | 90.3 | 78.7 | 93.5 | 75.4 | 82.3 | 52.5 | 56.5 |
| MCWC | 79.5 | 77.1 | 85.5 | 84.3 | 48.2 | 42.3 | 74.7 | 67.6 | 72.3 | 82.1 | 55.4 | 65.2 | 66.3 | 77.3 | 42.2 | 39.9 | 22.9 | 24.2 |
| UHC | 87.4 | 85.1 | 83.9 | 93.2 | 57.3 | 49.5 | 83.1 | 58.4 | 77.8 | 77.4 | 66.3 | 62.0 | 71.5 | 72.9 | 53.6 | 46.5 | 36.3 | 25.1 |
| UH &FWC | 30.4 | 16.7 | 37.6 | 42.6 | 14.1 | 4.0 | 36.6 | 41.8 | 24.1 | 55.0 | 16.5 | 35.9 | 29.0 | 45.6 | 10.6 | 2.6 | 0.5 | 1.2 |
| UnSC/RD | 36.2 | 16.8 | 45.7 | 38.1 | 9.0 | 2.5 | 53.9 | 47.2 | 56.0 | 61.8 | 33.6 | 35.5 | 44.5 | 48.6 | 9.0 | 1.4 | 7.9 | 0.4 |
| CC | 9.6 | 23.2 | 24.1 | 46.5 | 18.5 | 13.8 | 27.4 | 47.3 | 20.9 | 50.6 | 11.5 | 35.3 | 26.0 | 49.0 | 8.6 | 13.8 | 0.0 | 13.8 |
| NGO clinic/hospital | 72.4 | 74.7 | 66.9 | 74.1 | 39.0 | 42.7 | 58.5 | 51.9 | 65.3 | 60.4 | 52.2 | 41.4 | 58.7 | 64.7 | 36.6 | 35.8 | 27.6 | 9.0 |
| Private | 86.9 | 98.8 | 86.4 | 100.0 | 60.6 | 63.0 | 67.5 | 83.8 | 67.2 | 87.2 | 59.9 | 75.5 | 65.6 | 90.8 | 57.1 | 62.4 | 32.1 | 39.0 |
| **Division** |  |  |  |  |  |  |  |  |  |  |  |  |  |  |  |  |  |  |
| Barishal | 47.6 | 35.7 | 46.4 | 49.5 | 19.5 | 10.4 | 41.7 | 45.0 | 45.4 | 60.6 | 29.8 | 46.8 | 31.7 | 49.6 | 17.6 | 9.3 | 8.1 | 6.1 |
| Chattogram | 53.0 | 36.6 | 51.9 | 67.9 | 27.0 | 21.7 | 47.3 | 58.9 | 46.3 | 52.1 | 32.8 | 37.2 | 39.2 | 63.1 | 25.1 | 19.7 | 16.9 | 9.5 |
| Dhaka | 49.3 | 38.6 | 51.0 | 51.7 | 27.5 | 20.3 | 48.8 | 46.5 | 39.0 | 50.4 | 29.6 | 45.7 | 47.1 | 45.0 | 26.8 | 17.0 | 11.3 | 9.6 |
| Khulna | 41.5 | 38.0 | 57.6 | 56.1 | 34.4 | 18.4 | 42.7 | 42.1 | 37.5 | 79.0 | 29.0 | 44.0 | 57.7 | 48.2 | 27.2 | 16.9 | 10.8 | 10.3 |
| Rajshahi | 31.0 | 37.9 | 30.3 | 56.9 | 20.8 | 11.3 | 48.2 | 51.4 | 42.0 | 68.5 | 32.3 | 43.9 | 29.6 | 66.3 | 19.6 | 10.8 | 13.7 | 5.5 |
| Rangpur | 38.2 | 54.2 | 69.2 | 61.0 | 53.8 | 47.9 | 44.8 | 81.6 | 46.3 | 82.0 | 44.5 | 68.7 | 49.8 | 72.5 | 27.9 | 47.9 | 11.6 | 40.6 |
| Sylhet | 36.7 | 36.6 | 41.8 | 53.5 | 29.0 | 16.9 | 63.3 | 27.4 | 43.9 | 65.3 | 29.0 | 38.4 | 31.8 | 57.0 | 22.1 | 14.7 | 12.0 | 3.4 |
| Mymensingh |  | 26.4 |  | 46.5 |  | 11.1 |  | 25.7 |  | 54.1 |  | 21.1 |  | 39.8 |  | 11.1 |  | 5.5 |
| **Location** |  |  |  |  |  |  |  |  |  |  |  |  |  |  |  |  |  |  |
| Urban | 84.1 | 89.9 | 81.2 | 92.9 | 55.6 | 57.3 | 71.2 | 71.5 | 73.5 | 81.2 | 63.2 | 66.8 | 68.1 | 83.1 | 51.6 | 56.0 | 33.0 | 33.5 |
| Rural | 28.6 | 24.3 | 37.7 | 47.1 | 19.3 | 10.1 | 38.3 | 45.1 | 29.3 | 56.2 | 19.4 | 37.7 | 32.2 | 48.7 | 13.8 | 8.4 | 4.2 | 5.2 |
| **^1^Antibiotics, oxytocin, anticonvulsant** | | | | | | | | | | | | | | | | | | |
